# New Markers of Platelet Activation and Reactivity and Oxidative Stress Parameters in Patients Undergoing Coronary Artery Bypass Grafting

**DOI:** 10.1155/2021/8915253

**Published:** 2021-06-26

**Authors:** Petar Vukicevic, Aleksandra Klisic, Vojislava Neskovic, Luka Babic, Aleksandar Mikic, Natasa Bogavac-Stanojevic, Milos Matkovic, Svetozar Putnik, Nemanja Aleksic, Jelena Kotur-Stevuljevic

**Affiliations:** ^1^Clinic for Cardiac Surgery, Military Medical Academy, Belgrade, Serbia; ^2^University of Defense, Medical Faculty of the Military Medical Academy, Belgrade, Serbia; ^3^Primary Health Care Center, University of Montenegro-Faculty of Medicine, Podgorica, Montenegro; ^4^Clinic for Anesthesiology and Critical Care, Military Medical Academy, Belgrade, Serbia; ^5^Clinic for Cardiac Surgery, UC Clinical Centre, Belgrade, Serbia; ^6^University of Belgrade-Faculty of Medicine, Belgrade, Serbia; ^7^Department for Medical Biochemistry, University of Belgrade-Faculty of Pharmacy, Belgrade, Serbia; ^8^Department for Cardiac Surgery, Clinical Center of Serbia, Belgrade, Serbia

## Abstract

**Objective:**

Recent studies have shown that the red cell distribution width- (RDW-) to-platelet (PLT) count ratio (i.e., RPR) and the mean platelet volume (MPV)/PLT ratio (i.e. MPR) are more sensitive markers of atherosclerosis-connected risk than RDW and PLT alone. The present study is aimed at investigating the oxidative stress status and these two new markers of platelet activation in two different heart surgery modalities: cardiopulmonary bypass (CPB) and off-pump coronary artery bypass (OPCAB). We also aimed to test the possible relationship between RPR and MPR, respectively, and the severity and complexity of atherosclerotic plaque, measured as Syntax Score. *Patients and Methods*. A total of 107 patients encompassed this prospective study (i.e., 60 patients in CPB group and 47 patients in OPCAB). Blood samples were drawn at several time intervals: before skin incision (t1), immediately after intervention (t2), 6 h (t3), 24 h (t4), 48 h (t5), and 96 h after cessation of the operation (t6).

**Results:**

The values of RPR and MPR were similar in CPB and OPCAB before surgery and started to rise in t2 (i.e., immediately after the intervention). This increase lasted to t5 (i.e., 48 hours after the intervention), when it became the highest. After that, both markers started to regress about the 96^th^ hour after the beginning of surgery. Nominal values of both indices were higher in CPB than in OPCAB in all study points after the surgery. Furthermore, a significantly higher level of antioxidative parameters (i.e., total sulfhydryl groups and paraoxonase 1) in the OPCAB group compared to the CPB group was noted at t5 study point (i.e., 48 hours after the surgery), whereas no significant difference was noted in prooxidant levels (i.e., lipid hydroperoxides and advanced oxidation protein products) between these groups at this study point. MPR and RPR correlated positively with Syntax Score at several study points after the surgery completion. Syntax Score, MPR, and RPR showed good clinical accuracy in surgery-related complication prediction ((AUC = 0.736), 95^th^ CI (0.616-0.856), *P* = 0.003)).

**Conclusion:**

When combined, MPV, RDW, and platelet count, such as MPR and RPR, could be good predictors of coronary artery disease status, regarding the aspect of joint inflammation, oxidative stress, and thrombosis.

## 1. Introduction

Oxidative stress, inflammation, and thrombosis are mutually involved in the atherosclerosis development, and progression of this features is evident as disease becomes irreversible [1, 2]. Oxidative stress and chronic systemic low-level inflammation and neurohumoral activation could cause increase in heterogeneity of erythrocytes in circulation and influence platelet reactivity [3, 4]. Previous studies have investigated the relationship between several simple and routine hematological parameters and atherosclerotic plaque gravity and complexity prediction of unwanted acute cardiovascular events, and acute myocardial infarction outcome [3–6]. Decreased platelet (PLT) count connected with increased mean platelet volume (MPV) could predict changes in platelet reactivity and aggregability [6, 7]. On the other hand, red cell distribution width (RDW) per se is related with adverse outcome in acute coronary syndrome [8–10].

Platelet response in any particular situation depends on the current balance between proaggregatory stimuli and antiaggregatory substances and is additionally governed by many traditional atherosclerosis risk factors: dyslipidemia, hypertension, and smoking [7]. Anisocytosis, measured as RDW is a well-recognized predictor of unwanted clinical outcomes in different diseases, including coronary artery disease [11]. Increased RDW is in fact connected with several different aspects of red blood cells metabolic specificities. Namely, there are data about the influence of anemia, iron concentration and status, so as cholesterol membrane content on cells' deformability, shorter lifespan, and faster turnover, so all of these increase RDW, as a measure of erythrocyte activation upon united oxidative stress and inflammation conditions [8].

Also, erythrocytes may become a part of advanced atherosclerotic plaque, which on one side lowers these blood cells' count in systemic circulation and on the other side increases cholesterol content in the lipid core of the plaque, and thus directly influence its stability. Otherwise, lower red blood cells' count pushes immature cells into the bloodstream [9–11].

We have recently shown increased oxidative stress in patients before and after coronary artery bypass grafting (CABG) [12]. In order to get deeper insight into the complex pathophysiological mechanisms of coronary artery disease, we continued the quest for more sensitive markers connected with erythrocytes and platelets activation which is presumably governed by oxidative stress, inflammation, and advanced atherosclerosis condition. Recent studies have shown that the RDW-to-PLT count ratio (i.e. RPR) and the MPV/PLT ratio (i.e., MPR) are even more sensitive markers of atherosclerosis-connected risk [5, 6], than RDW and PLT alone. Also, previous studies have shown an inflammatory and thrombotic predictive role of MPV, RDW, MPR, and RPR in many other diseases, such as obesity, type 2 diabetes, and hepatosteatosis [13–16].

The present study was designed to estimate oxidative stress status and the two new markers of platelet activation and reactivity in the two different heart surgery modalities (cardiopulmonary bypass (CPB) and off-pump coronary artery bypass (OPCAB)). We also aimed to test the possible connection between these two hematological indices and severity and complexity of atherosclerotic plaque, measured as Syntax Score [17].

To the best of our knowledge, RPR and MPR have not been examined regarding CPB and OPCAB surgical procedures. We hypothesize that these two indices of platelet and erythrocyte activation in atherosclerotic setting could be increased as a consequence of long-term oxidative stress and inflammation influence.

This is an important issue, because larger disturbance in reductive balance during and after the cardiac surgery leads to poorer outcomes and increased mortality in operated patients.

## 2. Materials and Methods

### 2.1. Study Design and Patients

The study was planned as a prospective, interventional study at the Department for Cardiac Surgery, Medical Military Academy, Belgrade, Serbia. The whole study was planned according the ethical standards following the Declaration of Helsinki, as revised in 1983; the study protocol was approved by the institutional Ethics Committee, and all patients involved in the study signed an informed consent.

The study involved a total of 107 patients scheduled for an elective operation with one to four CABG. The patients were divided according to the number of bypass graft received during the CABG surgery, and also according to surgery modality, i.e., CPB (*n* = 60) and OPCAB (*n* = 47) subgroups. In the CPB group, the patients underwent surgery using CPB on the potassium-arrested heart, whereas in the OPCAB group, the patients underwent surgery on the beating-heart without a CPB machine.

Exclusion criteria for the current study patients were as follows: redo surgery or emergency surgery, concomitant valvular disease, ventricular aneurysms, myocardial infarction within the past 3 months or perioperative myocardial infarction, cerebral insult within the past 3 months, usage of systems for intraoperative blood salvage—Cell Saver machine and tubing system—systemic inflammatory or malignant disease, immunosuppressive drugs usage, massive postoperative mediastinal bleeding, heart failure, and presence of an infection.

Aspirin and clopidogrel were excluded from the therapy 7-10 days before the surgery. In both groups, all patients received low-molecular weight heparin (50-70 IU/kg), preoperatively.

### 2.2. Anesthesia

The anesthetic technique was standardized for all patients and consisted of balanced anesthesia. General anesthesia was induced by etomidate (0.1–0.2 mg/kg), and sufentanil (0.5–1 *μ*g/kg) and maintained with sevoflurane (0.6–1.0%) and sufentanil (0.5–1 *μ*g/kg/h).

### 2.3. Surgical Procedure

In both groups, midline sternotomy and harvesting of left internal mammary artery as a pedicle and saphenous vein grafts were followed by full exposure of the coronary artery branches to be revascularised.

### 2.4. Syntax Score Calculation

The Syntax Score [17] which is a broadly used tool for prediction events following percutaneous coronary intervention was obtained for each patient that underwent CABG.

### 2.5. Sample Collection and Analyses

Blood samples were collected at different time points according to the study protocol. Samples were obtained before skin incision (t1), immediately after protamine-sulfate administration (t2), 6 hours (t3), 24 hours (t4), 48 hours (t5), and 96 hours after cessation of operation and surgical trauma (t6). In all patients, the samples of venous blood were obtained from the central venous line from jugular internal vein. The venous blood collection was drawn into test tubes (Vaccuette® Blood Collection Tubes, Greiner Bio-One Diagnostics GmbH, Rainbach, Austria), and the sera were separated by centrifugation (Multifuge 3L, Heraeus, Kendro Laboratory Products, Osterode, Germany) and kept in 2 mL plastic tubes at −80°C until analysis.

Complete blood count results were obtained from the Cell-Dyn®3700 System, Abbott (Abbott Laboratories, IL, USA).

### 2.6. Oxidative Stress Status Markers

Parameters of oxidative stress were determined as described previously on an ILAB 650 analyzer (Instrumentation Laboratory, Milan, Italy). In brief, paraoxonase 1 (PON1) activity was determined kinetically with a substrate-paraoxon (Chem Service Inc., West Chester, Pennsylvania, USA) using the method of Richter and Furlong [18]. The total sulfhydryl groups' (tSHG) levels were determined using dinitrodithiobenzoic acid as a reagent in alkaline buffer referred to Ellman's method [19]. Advanced oxidation protein products (AOPP) were measured according to the method of Witko-Sarsat, by a reaction with potassium iodide and glacial acetic acid [20]. Lipid hydroperoxides (LOOH) were determined by the method of Gay and Gebicki, using the ferric-xylenol orange method, following the precipitation in perchloric acid [21].

### 2.7. RPR and MPR Indices Calculation

RPR is calculated as the ratio of RDW and PLT number, and MPR as MPV and PLT number.

### 2.8. Statistical Analysis

Parameters' distribution was estimated by the Shapiro-Wilk test and Kruskall-Wallis nonparametric analysis of variance, followed by the Mann-Whitney *U* test in order to assess intergroup differences. The comparison between categorical variables was performed by the Chi-square test. The correlation between variables was checked with using Spearman's nonparametric correlation method. Binary logistic regression analysis was used to investigate the association of Syntax Score values with RPR and MPR concentration increment 48 hours after the surgery (unadjusted and after the adjustment for starting values of the parameter of interest).

Clinical accuracy of the examined parameters was assessed by using the receiver operating characteristic (ROC) curve analysis towards postoperative complications presence.

Statistical analysis was performed with SPSS version 18.0 (IBM, Chicago, IL, USA). In all analyses *P* value < 0.05 was considered statistically significant.

## 3. Results

A total number of 60 patients were included in the CPB group, whereas 47 patients encompassed the OPCAB group. There were no difference in age and gender distribution between groups (*P* > 0.05).

Comparing the general and clinical characteristics of the two subgroups, we noticed a significantly higher body mass index (BMI) in the CPB group, higher percent of diabetic patients who used insulin, and also higher Syntax Score (Table 1). Other clinical variables were comparable in both study subgroups related to the surgery modality.

In order to get a more precise insight into the surgical condition influence on selected hematological parameters, the patients were categorized according to the surgical modality (CBP vs. OPCAB), and then in each of the two main groups, further division was performed according to the number of bypass grafts patients received during the surgical revascularisation procedure.

**Figure 1:**
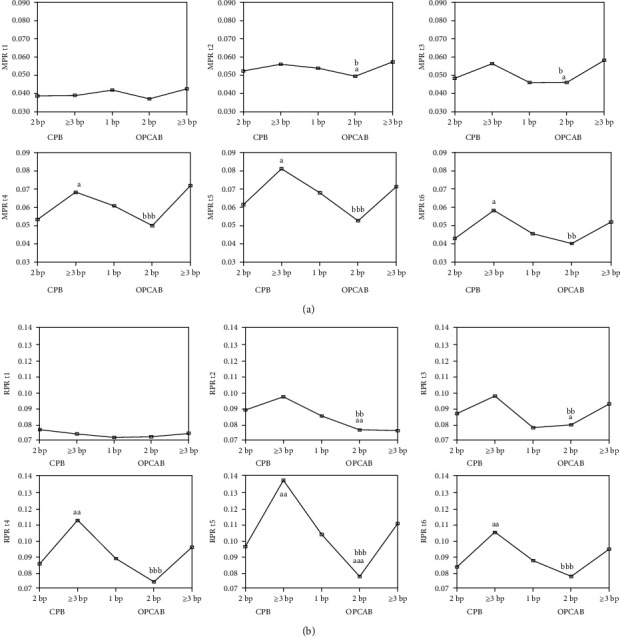
MPR (a) and RPR (b) according to bypass number and surgery modality during different study points. ^a^*P* < 0.05, ^aa^*P* < 0.01, and ^aaa^*P* < 0.001 vs. the 2 bp CPB group, ^b^*P* < 0.05, ^bb^*P* < 0.01, and ^bbb^*P* < 0.001 vs. the 3 bp CPB group. The Kruskal-Wallis test and then the Mann-Whitney *U* test.

Results of this part of analysis are presented in Figure 1.

Analysing this part of results, we could notice that both platelet markers (i.e., MPR and RPR) were increased during the surgery, but increment was always higher in the CPB compared to the OPCAB group, and also regarding the bypass graft number of subgroups (augmentation was increasing with the number of implemented bypass grafts). If we look at patients' division this way, it is obvious that the peak values for both indices are reached in t5 study point, i.e., 48 hours after the surgery.

The results of potential correlation between the MPR and RPR parameters and Syntax Score are presented in Table 2.

Our results revealed a significant positive correlation between the levels of atherosclerotic plaque complexity, estimated through the Syntax Score value and platelet activity indices, i.e., MPR and RPR in several study points after the completion of surgical procedure.

We have also measured several oxidative stress status markers, and here, we presented their values at t5 study point, since MPR and RPR reached the peak increment values at this study point.

**Figure 2:**
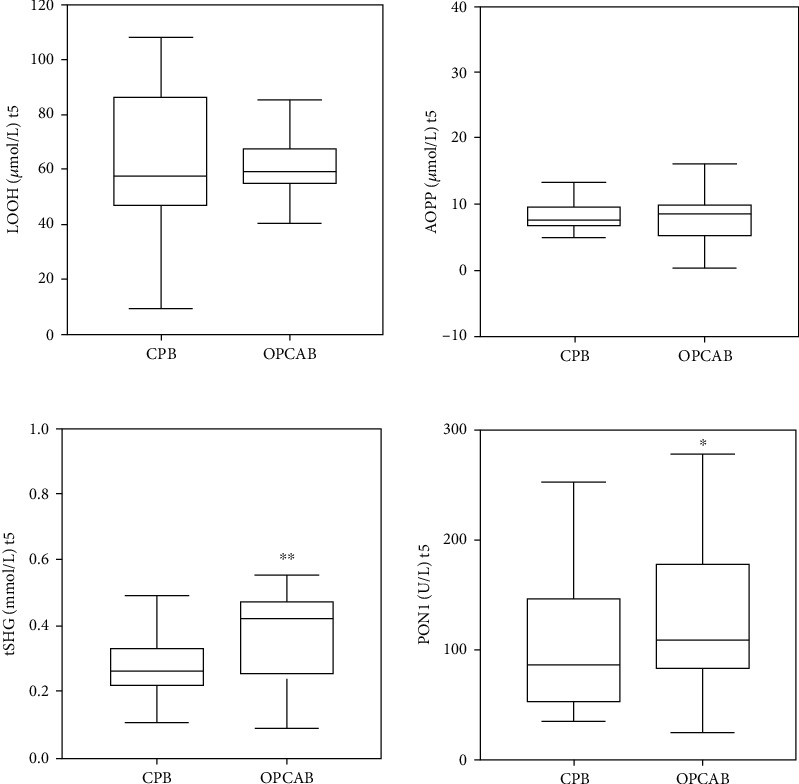
Oxidative stress status parameters at t5 (48 h after surgery), when platelet and erythrocyte indices showed the peak increment.

Figure 2 shows the concentration of LOOH, AOPP, and tSHG and the activity of PON1 at t5 (i.e., 48 hours after the surgery), when platelet and erythrocyte indices showed the peak increment.

Results showed significantly higher antioxidative parameters (i.e., tSHG and PON1) in the OPCAB group compared to the CPB group. Other oxidative stress status markers did not differ regarding implemented surgical technique 48 hours after the surgery completion.

Binary logistic regression analysis was performed in order to estimate the coronary vessel wall stenosis complexity influence (as Syntax Score value) on RPR and MPR amplification 48 hours after the surgery (study point when these indices reached the peak increment) (Table 3).

This analysis confirmed the significant influence of Syntax Score as a measure of plaque complexity on platelet activation indices, unadjusted, and even after the adjustment for basal RPR and/or MPR values, respectively. We also estimated the adjustment for oxidative stress parameter model (LOOH t5, AOPP t5, PON1 t5, and tSHG t5). Values of these parameters were measured in the same study point when platelet indices reached maximal increment (48 hours after the surgery). Oxidative stress parameters could not abrogate the Syntax Score influence at platelet activation indices increment, which further confirmed its independent influence of two calculated measures of platelet and erythrocyte activity and functionality.

We further performed ROC analysis to estimate possible clinical accuracy of these two new indices of platelet and erythrocyte function and activity, so as preoperative Syntax Score value in the prolonged time after the specific surgical treatment to predict clinical complication evolution in a group of CABG patients. It is important to note that complications were developed in 15 patients (i.e., 14% of all patients), mainly pulmonary thromboembolism, pleural effusion, shallow sternal wound infection, and leg wound infection.

**Figure 3:**
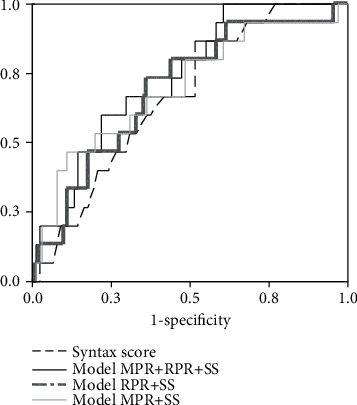
ROC curve for the postoperative complication prediction accuracy.

Results are presented in Figure 3 and Table 4.

We constructed logistic regression models in order to get more accurate complication prediction, and the results of ROC analysis showed that the combination of Syntax Score, MPR, and RPR (integrated values of all study times) showed the best accuracy, compared to other parameter combination (Table 4).

## 4. Discussion

Several important findings of the current study need to be mentioned. The values of both markers of platelet and erythrocyte-platelet-joined activation were similar in the two main patients' groups, CPB and OPCAB, and also in subgroups according to bypass number, before surgery. MPR and RPR started to rise in t2 (i.e., immediately after the intervention), and this increase lasted to t5 (i.e., 48 hours after the intervention), when it became the highest. After that, both markers started to regress about the 96^th^ hour after the beginning of surgery. It is important to note that nominal values of both indices were higher in CPB than in OPCAB in all study points after the surgery (Figure 1). Furthermore, it should be emphasized that a significantly higher level of antioxidative parameters (i.e., tSHG and PON1) in OPCAB group compared to CPB group was noted at t5 study point (i.e., 48 hours after the surgery), when platelet and erythrocyte indices showed peak increment.

Unal et al. [22] considered MPV a main marker of platelet production rate and activation, better than the platelet count alone. We assumed that MPR is even a more sensitive indicator of an eventual thrombotic event. This ratio takes into account not only the platelet enlargement but also their number which, if started to fall, could serve as a warning sign that platelets are actually wasting at thrombus formation.

The most convincing results of our current study which could explain the blood cells' involvement in atherosclerosis development is the significant correlation between MPR and RPR, respectively, with Syntax Score (Table 2). Syntax Score is a measure of atherosclerotic lesion severity and complexity [17], and here, we found positive correlation with platelet and erythrocyte-platelet activation indices at several study points after the surgery completion. To our knowledge, this is the first study which documented the direct relationship between the two platelet activity indices (i.e., MPR and RPR) and Syntax Score values. Several studies have already reported the relationship between MPV and RDW with different markers of atherosclerotic plaque severity, calculated as scores [23–25]. The work of Ekici et al. presented a positive correlation between MPV and two plaque related scores, i.e., Gensini and Syntax Score [23]. Willoughby et al. [7] explained the way of platelet activation upon atherosclerotic pathological events. In such conditions, where plaque exists in different phases of formation, maturation, and rupture, endothelial damage exposes subendothelial structures, and then, platelets respond promptly by attaching at vulnerable places.

A few studies reported a significant positive correlation between RDW and plaque severity and complexity measured with Syntax Score [24, 25]. Bujak et al. [11] proposed in its review article the possible mechanisms of RDW relationship with atherosclerotic lesion complexity and thus negative prognostic effects of higher RDW through its connection with inflammation, oxidative stress, vitamin D, and iron deficiency on bone marrow function.

In our current study, we have noticed a higher increase in platelet activation indices in CPB than in the OPCAB group. This could be explained by the results from the study of Koning and associates [26].

They found that CPB causes changes in human plasma which further jeopardizes endothelial barrier function, especially cell-cell integrity. At the same time, CPB usage causes increase in endothelial activation markers (P-selectin, vascular cell adhesion molecule-1, von Willebrand factor plasma concentrations, and an increase in the angiopoietin-2-to-angiopoietin-1 ratio). It is expectable that increase in platelet consumption at the site of plaque formation leads to larger platelets release from the bone marrow [21].

In line with this, although oxidative stress status markers (i.e., LOOH and AOPP) did not differ regarding the implemented surgery modality 48 hours after the intervention, diminished antioxidative protection, in terms of lower activity of PON1 and lower levels of tSHG [12, 27], was found in the CPB than in the OPCAB group 48 hours after the intervention. This could be attributed to the decreased ability of patients who underwent CPB to respond to the oxidative stress, as well as more severe conditions of the CPB intervention than OPCAB [12].

We performed binary logistic regression analysis to estimate coronary vessel wall stenosis complexity influence (as Syntax Score value) on RPR and MPR amplification 48 hours after the surgery, i.e., the study point when these indices reached the peak increment (Table 3). Syntax Score remained a significant predictor of MPR and RPR values even after adjustment for its starting values, before surgery, and for oxidative stress indices. This leads us to conclude that the relationship between stenosis level and gravity and blood cells' activation indices are independent and involve some basic mechanisms of atherosclerotic disease progression, and certainly conditions during the surgery setting.

ROC analysis enabled us to test the clinical accuracy of these two new indices of platelet and erythrocyte function and activity and Syntax Score value in the time after the specific surgical treatment. ROC enabled to predict the clinical complication evolution in the group of CABG patients (in 15 patients, i.e., in 14% of them, we have noticed complications). The most important clinical complications in our group of patients were as follows: shallow sternal wound infection, pulmonary thromboembolism, leg wound infection, and pleural effusion.

Our analysis confirmed that integrated model of Syntax Score, MPR, and RPR had good [28] clinical accuracy in surgery-related complication prediction which additionally established their mutual connection in advanced atherosclerosis patients.

This research was conducted as a single-center which is one of the limitations of the study. Another drawback is a relatively small number of patients who underwent CABG. Taking this into account, a new larger sample size and multicenter studies are necessary to validate the obtained results.

## 5. Conclusion

The results of the current study imply conclusion that it is worthy to calculate this simple and inexpensive platelet functionality indices as an auxiliary tool for possible unwanted coronary artery disease event prediction. MPV, RDW, and platelet count are readily available as routine parameters and, when combined, such as MPR and RPR, could be good predictors of coronary artery disease status, regarding the joint inflammation, oxidative stress, and thrombosis aspect.

## Figures and Tables

**Table 1:** General characteristics of the study groups.

Variable	CPB group *N* = 60	OPCAB *N* = 47	*P*
Age, years	63.6 ± 9.3	64.7 ± 9.3	ns
Gender (males/females), *n* (%)^∗^	48/12 (81/19)	32/15 (68/32)	ns
BMI (kg/m^2^)	28.9 ± 3.8	27.0 ± 3.3	0.006
Obesity^∗^	9/28/23	13/25/9	ns
Normal weight/overweight/obese, *n* (%)	(15/47/37)	(28/53/19)	
Hypertension, *n* (%)^∗^	52 (88)	41 (87)	ns
Diabetes mellitus, *n* (%)^∗^	23 (39)	11 (23)	ns
Hyperlipidemia, *n* (%)^∗^	40 (68)	30 (64)	ns
Chronic obstructive pulmonary disease, *n* (%)^∗^	9 (15)	3 (6)	ns
Smoking, *n* (%)^∗^	42 (71)	33 (70)	ns
Myocardial infarction, *n* (%)	33 (56)	28 (60)	ns
Peripheral arterial disease, *n* (%)	22 (37)	24 (51)	ns
Family history, *n* (%)∗	45 (76)	35 (74)	ns
*Clinical characteristics*			
EuroSCORE Logistic	6 (3-8)	6 (4-9)	ns
Left ventricular ejection fraction, %	50.9 ± 9.2	48.8 ± 10.8	ns
Syntax Score	32.2 ± 9.29	20.7 ± 7.9	<0.001
*Previous medications*			
*β*-Blockers, *n* (%)∗	57 (97)	44 (94)	ns
Angiotensin-converting enzyme inhibitors, *n* (%)^∗^	51 (86)	42 (89)	ns
Calcium antagonists, *n* (%)^∗^	18 (30)	18 (38)	ns
Nitrates, *n* (%)^∗^	54 (92)	40 (85)	ns
Statins, *n* (%)^∗^	42 (71)	33 (70)	ns
Oral antidiabetics, *n* (%)^∗^	13 (22)	9 (19)	ns
Insulin, *n* (%)^∗^	10 (17)	2 (4)	0.040
Diuretics, *n* (%)^∗^	19 (32)	17 (36)	ns

^∗^Comparison performed by the *χ*^2^ test.

**Table 2:** Correlation between platelet activity indices, MPR and RPR with Syntax Score in coronary artery bypass grafting patients.

Syntax Score			
Parameter	*ρ*	Parameter	*ρ*
MPR t1	–	RPR t1	–
MPR t2	–	RPR t2	0.229^∗^
MPR t3	0.350^∗∗∗^	RPR t3	0.374^∗∗∗^
MPR t4	0.321^∗∗^	RPR t4	0.340^∗∗∗^
MPR t5	0.329^∗∗^	RPR t5	0.374^∗∗∗^
MPR t6	0.282^∗∗^	RPR t6	0.316^∗∗^

*ρ*: Spearman's rho. ^∗^*P* < 0.05, ^∗∗^*P* < 0.01, ^∗∗∗^*P* < 0.001.

**Table 3:** Binary logistic regression analysis for the association of Syntax Score with RPR and MPR increase above the 75^th^ percentile value 48 hours after the surgery.

Syntax Score	Wald	Odds ratio	95^th^ CI	*P*
RPR 48 h,				
Unadjusted Syntax Score	7.38	1.068	1.018-1.120	0.007
Adjusted Syntax Score for RPR b.s.	6.64	1.068	1.016-1.122	0.010
Adjusted Syntax Score for oxidative stress parameter model	9.76	1.084	1.028-1.140	0.002
MPR 48 h,				
Unadjusted Syntax Score	8.83	1.079	1.026-1.135	0.003
Adjusted Syntax Score for MPR b.s.	9.38	1.093	1.032-1.156	0.002
Adjusted Syntax Score for oxidative stress parameter model	10.80	1.094	1.037-1.153	0.001

b.s.: before surgery.

**Table 4:** ROC curve parameters for postoperative complication prediction.

Test result variable(s)	AUC (SE)	95^th^ CI	*P*
Syntax Score	0.668 (0.065)	0.540-0.796	0.038
Model MPR+Syntax Score	0.700 (0.077)	0.550-0.851	0.013
Model RPR+Syntax Score	0.692 (0.071)	0.552-0.832	0.017
Model MPR+RPR+Syntax Score	0.736 (0.061)	0.616-0.856	0.003

ROC parameters: area under the curve (AUC), standard error (SE), 95^th^ confidence interval, and *P* for Syntax Score. Models: SS in combination with MPR, RPR, and all three parameters.

## Data Availability

The data will be available upon reasonable request (contact person: aleksandranklisic@gmail.com).
